# Indomethacin Reduces Glomerular and Tubular Damage Markers but Not Renal Inflammation in Chronic Kidney Disease Patients: A Post-Hoc Analysis

**DOI:** 10.1371/journal.pone.0037957

**Published:** 2012-05-25

**Authors:** Martin H. de Borst, Ferdau L. Nauta, Liffert Vogt, Gozewijn D. Laverman, Ron T. Gansevoort, Gerjan Navis

**Affiliations:** 1 Division of Nephrology, Department of Internal Medicine, University Medical Center Groningen and University of Groningen, Groningen, the Netherlands; 2 Department of Nephrology, Academic Medical Center, Amsterdam, the Netherlands; Rouen University Hospital, France

## Abstract

Under specific conditions non-steroidal anti-inflammatory drugs (NSAIDs) may be used to lower therapy-resistant proteinuria. The potentially beneficial anti-proteinuric, tubulo-protective, and anti-inflammatory effects of NSAIDs may be offset by an increased risk of (renal) side effects. We investigated the effect of indomethacin on urinary markers of glomerular and tubular damage and renal inflammation. We performed a post-hoc analysis of a prospective open-label crossover study in chronic kidney disease patients (*n = 12*) with mild renal function impairment and stable residual proteinuria of 4.7±4.1 g/d. After a wash-out period of six wks without any RAAS blocking agents or other therapy to lower proteinuria (untreated proteinuria (UP)), patients subsequently received indomethacin 75 mg BID for 4 wks (NSAID). Healthy subjects (*n = 10*) screened for kidney donation served as controls. Urine and plasma levels of total IgG, IgG4, KIM-1, beta-2-microglobulin, H-FABP, MCP-1 and NGAL were determined using ELISA. Following NSAID treatment, 24 h -urinary excretion of glomerular and proximal tubular damage markers was reduced in comparison with the period without anti-proteinuric treatment (total IgG: UP 131[38–513] vs NSAID 38[17–218] mg/24 h, p<0.01; IgG4: 50[16–68] vs 10[1–38] mg/24 h, p<0.001; beta-2-microglobulin: 200[55–404] vs 50[28–110] ug/24 h, p = 0.03; KIM-1: 9[5]–[14] vs 5[2]–[9] ug/24 h, p = 0.01). Fractional excretions of these damage markers were also reduced by NSAID. The distal tubular marker H-FABP showed a trend to reduction following NSAID treatment. Surprisingly, NSAID treatment did not reduce urinary excretion of the inflammation markers MCP-1 and NGAL, but did reduce plasma MCP-1 levels, resulting in an increased fractional MCP-1 excretion. In conclusion, the anti-proteinuric effect of indomethacin is associated with reduced urinary excretion of glomerular and tubular damage markers, but not with reduced excretion of renal inflammation markers. Future studies should address whether the short term glomerulo- and tubulo-protective effects as observed outweigh the possible side-effects of NSAID treatment on the long term.

## Introduction

Reduction of proteinuria is a major therapy target to achieve long-term renoprotection in chronic kidney disease. To this end, blockade of the renin-angiotensin-aldosterone system (RAAS) ideally combined with a diuretic or dietary sodium restriction [1] is the cornerstone of current renoprotective therapy in chronic kidney disease [2], [3]. Nevertheless, chronic kidney disease (CKD) progression persists in a considerable proportion of patients. Particularly in patients with high residual proteinuria despite RAAS blockade, there may be a place for alternative modes of antiproteinuric intervention, such as non-steroidal anti-inflammatory drugs (NSAIDs) [4].

Before the era of RAAS inhibitors, NSAIDs were well known for a distinct antiproteinuric effect. Their use in nephrotic syndrome was supported by retrospective data suggesting that NSAIDs may retard progression of renal function loss [5], [6], although not all data are uniform [7].

Most renoprotective effects of NSAIDs are considered to be related to their effect on prostaglandin synthesis [8]. Prostaglandin inhibition reduces effective renal plasma flow (ERPF) and glomerular filtration rate (GFR), presumably resulting in a reduction of intraglomerular pressure, in turn leading to a reduction of proteinuria [9]. Moreover, through inhibition of prostaglandin synthesis, NSAIDs reduce inflammation, a determinant of renal and cardiovascular outcome in CKD [10], [11]. Recent elegant studies implicated a central role for increased cyclooxygenase-2 (COX-2) expression in podocytes in the etiology of glomerular injury which could be reversed by COX-2 inhibition [12], [13]. Beside these beneficial effects, however, NSAIDs also have side-effects in several organ systems including the gastro-intestinal tract (e.g. peptic ulcers) and central nervous system side-effects. Also deleterious renal effects such as tubulointerstitial damage have been reported [14], [15]. Since the presence of tubulointerstitial damage is a determinant of response to renoprotective therapy [11], this may negatively impact prognosis for CKD patients.

To further investigate the glomerular, tubular and anti-inflammatory effects of NSAIDs in CKD, we measured a panel of urinary renal damage markers representing different parts of the nephron in patients with overt proteinuria, before and after treatment with indomethacin. As a secondary aim we investigated whether pretreatment biomarker levels were associated with the anti-proteinuric response.

## Materials and Methods

### Ethics Statement

The study was approved by the medical ethics committee of the University of Groningen, and all participants provided written informed consent. All clinical investigations have been conducted according to the principles expressed in the Declaration of Helsinki.

### Patients

This study is performed as a post-hoc analysis of a prospective open-label crossover study [16]. This study was performed in Caucasian patients (n = 16) who fulfilled the following inclusion criteria after a 6 week washout period without RAAS intervention: proteinuria >2 g/day, diastolic blood pressure <90 mmHg, creatinine clearance >30 mL/min and age 18–70 years. Blood and urine samples were available for 12 out of 16 patients due to biobank exhaustion: seven patients with non-diabetic nephropathy (membranous glomerulopathy (n = 2), primary focal segmental glomerular sclerosis (2), IgA nephropathy (2) and hypertensive nephropathy (1)), and five with (type 2) diabetic nephropathy. Diagnoses were established by kidney biopsy for all patients. The characteristics of these patients were similar to the original study population. Healthy subjects who were screened for kidney donation in our center were included as controls (n = 10).

### Study Protocol

The detailed study protocol was published previously [16]. During an initial washout period, blood pressure was titrated with hydrochlorothiazide 12.5 mg QD combined with amlodipine or doxazosine if necessary; this regimen remained unchanged during the actual study. Following the washout period without anti-proteinuric treatment, urine and blood samples were collected. Subsequently patients were treated with indomethacin 75 mg BID (retard formula; Indocid® Merck & Co., Inc., Whitehouse Station, NJ, USA) for four weeks. At the end of this period a second set of urine and blood samples were collected.

### Clinical Measurements

At each visit, after an overnight fast, blood was sampled, 24-h urine was collected and blood pressure was measured by an automatic device (Dinamap®, GE Healthcare, Waukesha, WI, USA). Twentyfour-hour urine was checked for collection errors using the equation previously formulated by Ix et al [17]. The mean arterial blood pressure (MAP) was calculated as 2/3× diastolic blood pressure +1/3× systolic blood pressure. The mean value of four readings after 15 min was used for analysis. Urinary protein was determined with the pyrogallol red-molybdate method. Serum creatinine, albumin and lipids were determined using an automated multi-analyzer (MEGA®, Merck, Darmstadt, Germany).

### Measurement of Damage Markers

Aliquots from 24 h -urine were stored at −80°C until damage marker analysis. After thawing, all urine samples were vortexed and subsequently centrifuged (14.000 rpm). The supernatant was used for measurements. Samples were diluted to obtain optimal concentration for measurement. All urinary biomarkers were determined in one run. As glomerular damage markers we measured total IgG and IgG4, as markers of proximal tubular damage kidney injury molecule-1 (KIM-1) and beta-2-microglobulin (B2M), as markers of distal tubular damage heart-fatty acid binding protein (H-FABP), and as markers of inflammation neutrophil gelatinase associated lipocalin (NGAL), monocyte chemotactic protein-1 (MCP-1), as documented previously [18]. Urinary concentrations of total IgG, IgG4, NGAL, KIM-1, and H-FABP were measured by enzyme-linked immunosorbent assay. For KIM-1, B2M and NGAL, antibodies were obtained from R&D Systems (Minneapolis, MN, intra-assay CV 7.4%, 9.7% and 6.8%, respectively). H-FABP and total IgG, IgG-4 antibodies were obtained from Hytest (Turku, Finland, intra-assay CV 9.3%, 13.3% and 14.0%, respectively). All samples were measured in duplicate.

The 24-h excretion of renal damage markers was calculated by multiplying the urinary biomarker concentration with the total 24 h urine volume. Fractional excretion of renal damage markers was calculated as:
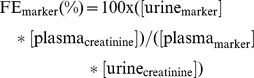



### Statistical Analyses

Data are expressed as median (interquartile range). Differences between healthy controls and CKD patients without anti-proteinuric treatment were analyzed by Mann Whitney non-parametric test. Differences between the study periods without anti-proteinuric treatment versus NSAID treatment, respectively, were analyzed by Wilcoxon paired non-parametric test. Associations between urinary biomarker excretion and proteinuria were studied using linear regression with proteinuria as an independent variable, and the individual renal damage markers as dependent variables. For these analyses, urinary biomarker excretions were logarithmically transformed. We also determined whether the change in biomarker excretion between the study period without anti-proteinuric treatment and NSAID (delta biomarker) was related to the anti-proteinuric response (delta proteinuria). Furthermore similar analysis were performed for the association between biomarker excretion during the period without anti-proteinuric therapy (i.e. before anti-proteinuric intervention) and the anti-proteinuric response, to evaluate which biomarker(s) would be able to predict the anti-proteinuric response. For all analyses, a two-sided p<0.05 was considered statistically significant.

All calculations and analyses were performed using SPSS 18.0 for Windows (SPSS Inc., Chicago, Illinois, USA).

## Results

**Figure 1:**
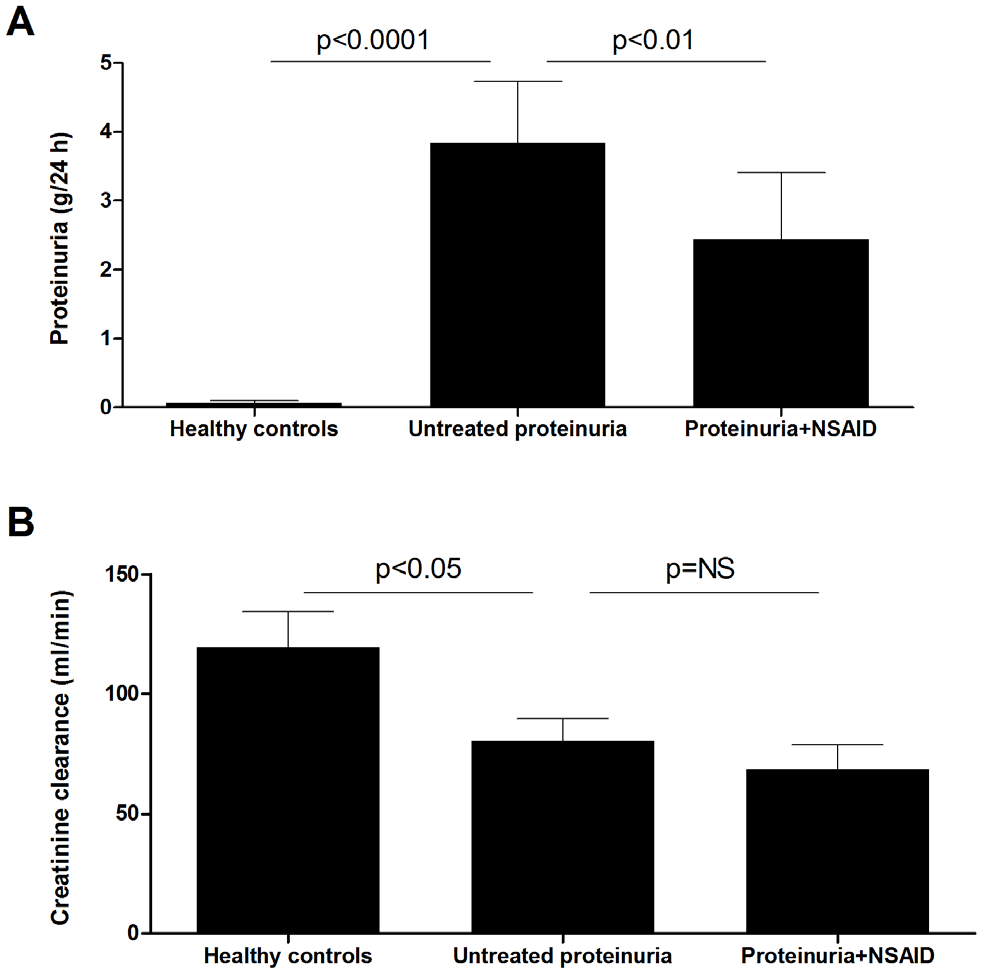
Proteinuria and creatinine clearance. A. Proteinuria was strongly increased in proteinuric CKD patients as compared to matched healthy controls. NSAID treatment significantly reduced proteinuria. B. Creatinine clearance was significantly lower in untreated CKD patients, whereas NSAID treatment did not significantly affect creatinine clearance. Horizontal lines indicate medians.

Baseline characteristics are given in Table 1. In CKD patients, proteinuria was reduced by NSAID treatment with a median of 51% (IQR 33–73%) compared with the study period without anti-proteinuric treatment (“untreated UP”; Figure 1A). CKD patients had a significantly lower creatinine clearance compared to healthy controls. After four weeks of treatment with indomethacin, creatinine clearance showed a non-significant trend to further reduction (Figure 1B).

**Table 1:** Baseline characteristics of proteinuric CKD patients and healthy controls.

	Healthy controls	CKD untreated	CKD+NSAID
n	10	12	12
Age (years)	57 (41–68)	56 (42–67)	56 (42–67)
Male sex (%)	70	69	69
Diabetes mellitus (n)	0	5*	5
Systolic blood pressure (mmHg)$	119 (105–151)	142 (125–179)*	148 (130–165)
Diastolic blood pressure (mmHg)$	70 (55–84)	81 (72–95)*	79 (74–85)
BMI (kg/m^2^)	27 (23–29)	28 (23–38)	28 (25–34)
Body weight (kg)	87 (74–97)	89 (76–101)	88 (77–105)
Creatinine clearance (ml/min)	113 (59–187)	79 (30–155)*	70 (28–140)
Proteinuria (g/24 h)	0.0 (0.0–0.2)	3.3 (0.6–14.6)**	1.2 (0.7–2.5)^##^
UNaV (mmol/24 h)	219 (148–266)	213 (127–253)	173 (109–210)#
FE sodium (%)	0.9 (0.6–1.3)	1.5 (1.2–3.5)**	1.5 (1.1–2.9)

BMI, body mass index; UNaV, urinary sodium excretion; FE, fractional excretion.

* p<0.05, **p<0.01 CKD untreated vs healthy controls.

# p<0.05, ^##^ p<0.01 CKD NSAID vs CKD untreated.

$ Antihypertensive use remained stable during the study protocol.

In parallel with proteinuria, 24-h urinary excretions of the glomerular damage markers total IgG and IgG4 were strongly increased in CKD patients as compared to healthy controls. Anti-proteinuric treatment with indomethacin reduced urinary total IgG and IgG4 excretion (Table 2). Plasma levels of total IgG and IgG4 were not affected by NSAID treatment (Table S1); therefore fractional excretion of these markers decreased significantly during treatment (Table 3). The glomerular charge selectivity index, calculated as the ratio of the fractional excretions of total IgG/IgG4, increased with NSAID treatment (untreated proteinuria: 6.98[1.24–16.8] vs NSAID 9.96[2.02–30.92], p<0.05).

**Table 2:** 24-h urinary excretion of renal damage markers in healthy controls, CKD patients after a period with no anti-proteinuric treatment (untreated UP), and after NSAID treatment.

Damage marker	Healthy controls	Untreated UP	P vs healthy	NSAID	P vs untreated UP
**Total IgG (mg/24 h)**	3.9 (2.9–5.6)	130.9 (37.8–513.4)	<0.001	38.1 (17.0–218.4)	<0.01
**IgG4 (mg/24 h)**	0.0 (0.0–0.1)	50.3 (16.4–68.3)	<0.001	10.2 (1.2–38.0)	<0.001
**KIM-1 (µg/24 h)**	2.3 (1.6–4.9)	8.9 (5.2–13.9)	0.004	4.6 (2.0–7.8)	0.01
**B2M (µg/24 h)**	56.3 (34.8–75.6)	199.5 (54.9–404.2)	0.03	47.8 (27.6–109.6)	0.03
**H-FABP (µg/24 h)**	0.0 (0.0–0.2)	28.4 (8.7–11.9)	<0.001	9.1 (1.2–89.9)	0.11
**MCP-1 (µg/24 h)**	0.8 (0.4–1.1)	1.8 (0.9–2.6)	0.01	1.8 (1.1–2.3)	0.88
**NGAL (µg/24 h)**	23.0 (15.1–29.4)	37.2 (29.2–89.9)	0.001	35.2 (26.8–89.9)	0.37

**Table 3:** Fractional excretion of damage markers in CKD patients after a period with no anti-proteinuric treatment, and after NSAID treatment.

Damage marker	Untreated UP	NSAID	P
**Total IgG (%)**	6.6×10^−4^(0.9–14.3×10^−4^)	3.0×10^−4^(0.7–8.0×10^−4^)	0.03
**IgG4 (%)**	1.25 (0.62–3.89)	0.31 (0.14–0.50)	0.004
**KIM-1 (%)**	4.70 (3.53–8.56)	3.16 (1.32–6.02)	0.03
**B2M (%)**	0.83 (0.19–11.13)	0.30 (0.14–2.83)	0.04
**H-FABP (%)**	24.9 (9.9–150.5)	22.7 (2.9–164.0)	0.72
**MCP-1 (%)**	5.61 (4.25–8.31)	13.23 (6.80–22.47)	0.006
**NGAL (%)**	1.60 (1.04–4.01)	1.73 (0.65–4.64)	0.40

The two proximal tubular markers KIM-1 and B2M were elevated in CKD patients during the study period without anti-proteinuric treatment, as compared to healthy controls, and reduced by NSAID treatment (Table 2). Fractional excretion of these markers was also significantly reduced by NSAID treatment (Table 3). Urinary excretion of the distal tubular marker H-FABP was elevated in untreated proteinuric patients when compared to healthy controls, and NSAID treatment resulted in a non-significant trend to reduced H-FABP excretion (Table 2) and a significant decline in plasma H-FABP levels (Table S1). Consequently the fractional excretion of H-FABP remained unchanged.

We subsequently investigated whether the anti-inflammatory drug indomethacin would affect renal inflammation, as reflected by urinary MCP-1 and NGAL excretion. Surprisingly, indomethacin did not reduce the 24 h -urinary excretions of MCP-1 or NGAL, both of which were higher in CKD patients than in healthy subjects (Table 2). In plasma, however, MCP-1 levels but not NGAL levels were reduced by indomethacin (Table S1). As a result, the fractional excretion of MCP1 increased (Table 3).

The relationship between urinary renal damage marker excretions and proteinuria was further investigated by regression analysis. Univariate analysis indicated that urinary excretions of total IgG (standardized beta.691, p<.001), IgG4 (standardized beta.642, p<.001), KIM-1 (standardized beta.662, p<.001), B2M (standardized beta.466, p<.05), and H-FABP (standardized beta.454, p<.05) were significantly associated with proteinuria. As expected, delta urinary total IgG excretion (with vs without antiproteinuric treatment, standardized beta.739, p<.01) and delta urinary IgG4 excretion (standardized beta.721, p<.01) were significantly associated with the anti-proteinuric response.

**Figure 2:**
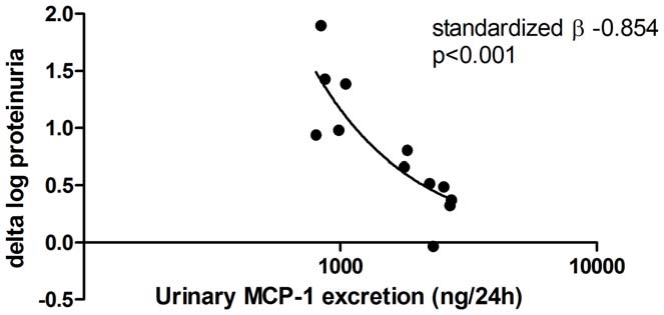
Pre-treatment urinary MCP-1 excretion and treatment response. Scatter plot indicating the relationship between the 24 h -urinary excretion of MCP-1 before start of anti-proteinuric therapy (i.e. NSAID) and the anti-proteinuric response (i.e. delta proteinuria without vs with NSAID). A negative association is present, suggesting that high baseline MCP-1 excretion is associated with poor anti-proteinuric response to therapy.

Finally we studied whether renal damage marker excretion during the period without anti-proteinuric treatment (i.e. before anti-proteinuric therapy) would be associated with the anti-proteinuric response to indomethacin. Interestingly, urinary excretion of KIM-1 (standardized beta −0.673, p<0.05) and MCP-1 (standardized beta −0.854, p<0.001) before anti-proteinuric treatment was inversely associated with the anti-proteinuric response. After adjustment for age and genderonly the association between MCP-1 and the anti-proteinuric response remained significant (standardized beta −0.662, p<0.01). A scatter plot illustrating the relationship between pre-treatment MCP-1 levels and the anti-proteinuric response is presented in Figure 2.

## Discussion

RAAS blockade combined with volume restriction is the therapy of choice for most CKD patients, but the anti-proteinuric response to this regimen is often incomplete [19]. Therefore it is crucial to identify interventions that can serve as (adjunct) anti-proteinuric therapies. The main finding of the current study is that the anti-proteinuric effect of the NSAID indomethacin is accompanied by reduced urinary 24 h -excretion of glomerular and tubular damage markers. The 24 h -urinary excretion of inflammation markers, on the other hand, was not affected by NSAID treatment, and the fractional excretion of MCP-1 was even increased.

The reduced urinary excretion of glomerular damage markers total IgG and IgG4 is in line with the known anti-proteinuric effect of indomethacin, and may be related to prostaglandin inhibition affecting renal hemodynamics (i.e. vasoconstriction of the afferent arterioles). Furthermore, both 24 h -urinary excretion and fractional excretion of the proximal tubular damage markers were reduced following treatment with an NSAID, and the distal tubular marker H-FABP showed a trend to reduction. This suggests that NSAIDs may have protective effects, rather than toxic effects, on the tubular epithelium. Tubulotoxic effects may be seen in rare cases, but in our study on average signs of reduction of tubular damage are observed, probably secondary to the anti-proteinuric effect of NSAID treatment. This is in concert with previously observed tubulo-protective effects of RAAS-related anti-proteinuric therapy in both clinical cohorts [20] as well as preclinical studies, where also an association with protection against structural renal damage was observed [21], [22]. In addition, direct protective (e.g. anti-apoptotic) effects as implicated by in vitro studies [15] may play a role. NSAID treatment did not significantly affect the distal tubular marker H-FABP, although a trend to reduction was observed. Much less is known about the effects of proteinuria on distal as compared to proximal tubular cells, although the distal tubule is considered less sensitive to the toxic effects of proteinuria. We recently reported that H-FABP is associated with albuminuria in diabetic subjects; moreover, H-FABP was the only damage marker from the investigated marker set that was significantly associated with eGFR after adjustment for albuminuria [18]. Future studies should address the relevance of H-FABP modulation by antiproteinuric therapy.

Surprisingly, NSAID treatment had no effect on the 24 h-urinary excretion of inflammation markers MCP-1 or NGAL. In contrast, fractional excretion of MCP-1 even increased following NSAID treatment. This was unexpected given the anti-inflammatory properties of this drug. On the other hand, the dissociation of renoprotective effects from anti-inflammatory effects of NSAIDs is in line with preclinical studies [15], [23]. Furthermore, we recently found that in a cohort of CKD patients receiving an angiotensin receptor blocker in addition to an angiotensin converting enzyme inhibitor, urinary excretion of proximal tubular markers was reduced, but inflammatory markers did not change (*data submitted*). Our finding that the extent of pre-treatment renal inflammation is independently associated with the anti-proteinuric response is also in line with preclinical studies [11]. This finding may have two important implications. First, these data suggest that the renoprotective potential of NSAIDs may be limited by the absence of intra-renal anti-inflammatory effects (in spite of systemic anti-inflammatory effects as reflected by lower plasma MCP-1 and B2M levels). Second, these data suggest that the antiproteinuric, and potentially also the renoprotective effect of NSAIDs is limited to disease states with less intra-renal inflammation, for instance excluding the use of these drugs in immune-mediated renal disease.

Treatment with indomethacin induced a trend towards decreased creatinine clearance in our study, and although this reduction was relatively small, our study lacks power to exclude an effect on renal function. Previous studies demonstrated an anti-proteinuric response in the presence of a significant decline of GFR following NSAID treatment [24], [25]. The limited decline in creatinine clearance in our study may also be explained by the sodium replete state of our population. Although dietary salt restriction is part of current best available renoprotective therapy, in clinical practice it is notoriously difficult to implement a persistent reduction in dietary salt intake, and in fact in most published studies, the salt intake of CKD patients is similar to that in the general population [26] [26]. In line, several landmark randomized controlled trials published over the last years have also not been performed against the background of dietary sodium restriction. In the setting of NSAID treatment, however, sodium restriction may increase the risk of a fall in GFR [4]. Of note, the antiproteinuric effect of NSAIDs has also been described to be blunted by sodium repletion at least in severely nephrotic patients [27]. The current anti-proteinuric efficacy of NSAID treatment despite high sodium intake is therefore remarkable, suggesting an optimal balance between efficacy and safety of NSAIDs under liberal sodium intake. Our findings also suggest that NSAID treatment increased charge selectivity, which is known to be reduced in nephrotic-range proteinuria [28].

NSAIDs are nowadays rarely considered for anti-proteinuric therapy in CKD. This is for the large part explained by the availability of RAAS blockers with a more favorable potential, whereas NSAIDs may have tubulotoxic effects. Also, the reduction of creatinine clearance observed in up to 20% of patients treated with NSAIDs [29], [30], although reversible and presumably reflecting a beneficial renal hemodynamic effect (lowering of intraglomerular pressure), has raised concern against using these drugs in patients with CKD. In our study, none of the 24 h-excretions of renal damage markers increased following NSAID treatment as compared to the period without anti-proteinuric treatment. The decrease in creatinine clearance was small and did not reach statistical significance, perhaps due to the sodium-replete condition of our patients. The limited follow-up of our study does not allow to address longer term safety of NSAID therapy.

Our study has its limitations such as limited sample size and study duration, and the post-hoc design. Given the heterogeneity of the population with respect to the underlying type of renal disease, it may be surprising that significant reductions of glomerular and proximal tubular biomarkers were found. This suggests that irrespective of the underlying disease, one or more shared pathophysiologic pathways (including proteinuria) are reduced by NSAID treatment. The current findings cannot be extrapolated to patients with more severe renal disease, who may experience more side effects such as hyperkalemia or edema, or patients on background RAAS blockade. The latter category may not tolerate the combined regimen, and a previous study has questioned the additional efficacy of NSAID when give on top of maximum RAAS blockade as anti-proteinuric therapy [31]. Furthermore it would be of interest to study intra-individual changes of these biomarkers over time, and their prognostic value, in a longitudinal analysis. Nevertheless our data may provide a rationale to prescribe NSAIDs in patients with glomerular disease and proteinuria in whom immunosuppressive therapy and RAAS blockade are either ineffective or contra-indicated. Randomized controlled clinical trials investigating the long-term renoprotective (i.e. protective against loss of renal function) as well as cardiovascular effects of NSAIDs (as monotherapy or on top of RAAS blockade) are, however, warranted.

In conclusion, NSAID treatment is associated with a change in urinary biomarker profile that suggests glomerulo- and tubulo-protective effects along with reduction of proteinuria. Our findings, although they cannot be extrapolated to a setting of (maximally tolerated) background RAAS blockade with optimal volume depletion, suggest that under specific conditions NSAIDs may be used to reduce proteinuria as well as glomerular and tubular damage markers in CKD. This may be relevant given the prognostic importance of tubulointerstitial damage for the progression of renal function loss [32], [33]. On the other hand no intra-renal anti-inflammatory effects of NSAID treatment could be demonstrated and renal inflammation, another important prognostic determinant of renal function loss [34], may even be increased by NSAID treatment. NSAID may thus be considered with caution as a non-hypotensive antiproteinuric therapy when RAAS blockade is not tolerated, e.g. due to hypotension. Whether NSAIDs, either as monotherapy or given on top of RAAS blockade, have the potential to modulate long term renal outcome remains to be proven.

## Supporting Information

Table S1 Plasma levels of renal damage markers in CKD patients after a period with no anti-proteinuric treatment, and after NSAID treatment.
(DOC)
