# Supplementary material for: Indomethacin Reduces Glomerular and Tubular Damage Markers but Not Renal Inflammation in Chronic Kidney Disease Patients: A Post-Hoc Analysis
Source: PLoS One. 2012 May 25;7(5):e37957. doi: 10.1371/journal.pone.0037957 (PMC3360674; doi:10.1371/journal.pone.0037957)
Supplement: Table S1 — Plasma levels of renal damage markers in CKD patients after a period with no anti-proteinuric treatment, and after NSAID treatment. (DOC) [file pone.0037957.s001.doc]

**Table S1. Plasma levels of renal damage markers in CKD patients after a period with no anti-proteinuric treatment, and after NSAID treatment**

| **Damage marker** | **Untreated UP** | **NSAID** | **P** |
| --- | --- | --- | --- |
| **Total IgG (g/l)** | 23.9 (22.4-25.2) | 21.9 (18.9-23.8) | 0.12 |
| **IgG4 (mg/l)** | 0.18 (0.14-0.26) | 0.35 (0.14-0.52) | 0.89 |
| **KIM-1 (ug/l)** | 0.9 (0.0-1.4) | 0.0 (0.0-1.9) | 0.61 |
| **B2M (ug/l)** | 177 (121-324) | 105 (70-180) | 0.02 |
| **H-FABP (ug/l)** | 1.3 (0.2-3.1) | 0.5 (0.0-1.8) | 0.04 |
| **MCP-1 (ug/l)** | 0.21 (0.15-0.26) | 0.12 (0.09-0.13) | 0.003 |
| **NGAL (ug/l)** | 24 (20-26) | 25 (19-26) | 0.73 |
